# Dengue virus activates cGAS through the release of mitochondrial DNA

**DOI:** 10.1038/s41598-017-03932-1

**Published:** 2017-06-15

**Authors:** Bo Sun, Karin B. Sundström, Jun Jie Chew, Pradeep Bist, Esther S. Gan, Hwee Cheng Tan, Kenneth C. Goh, Tanu Chawla, Choon Kit Tang, Eng Eong Ooi

**Affiliations:** 1 Programme in Emerging Infectious Diseases, Duke-NUS Medical School, Singapore, 169857 Singapore; 2 Duke University School of Medicine, Durham, NC 27710 USA; 3 Department of Microbiology and Immunology, Yong Loo Lin School of Medicine, National University of Singapore, Singapore, Singapore; 4 Saw Swee Hock School of Public Health, National University of Singapore, Singapore, Singapore; 5 Singapore MIT Alliance in Research and Technology, Infectious Diseases Interdisciplinary Group, Singapore, Singapore

## Abstract

Cyclic GMP-AMP synthetase (cGAS) is a DNA-specific cytosolic sensor, which detects and initiates host defense responses against microbial DNA. It is thus curious that a recent study identified cGAS as playing important roles in inhibiting positive-sense single-stranded RNA (+ssRNA) viral infection, especially since RNA is not known to activate cGAS. Using a dengue virus serotype 2 (DENV-2) vaccine strain (PDK53), we show that infection creates an endogenous source of cytosolic DNA in infected cells through the release of mitochondrial DNA (mtDNA) to drive the production of cGAMP by cGAS. Innate immune responses triggered by cGAMP contribute to limiting the spread of DENV to adjacent uninfected cells through contact dependent gap junctions. Our result thus supports the notion that RNA virus indirectly activates a DNA-specific innate immune signaling pathway and highlights the breadth of the cGAS-induced antiviral response.

## Introduction

Pattern recognition receptors (PRRs) are integral components of the innate immune response and function to detect various pathogen components. For example, lipopolysaccharide from the Gram-negative bacterial cell wall activates toll-like receptor 4 (TLR4) while viral RNA can activate TLR3, TLR7, or RIG-I like receptors including RIG-I and MDA5^1^. Activation of these PRRs leads to nuclear translocation of transcription factors such as IRF3 and IRF7 and subsequent induction of type-I interferon (IFN-I) production^2^.

More recently, cGAS was identified as a PRR for cytoplasmic DNA^3^. When a DNA substrate binds cGAS, ATP and GTP substrates are used to generate cGAMP (cyclic GMP-AMP), which subsequently binds stimulator of interferon genes (STING). STING then activates and promotes the nuclear translocation of IRF3, leading again to production of IFN-I and activation of interferon stimulated genes (ISGs)^4^. Hence, introduction of nuclear DNA or DNA from pathogens such as bacteria or viruses into cytoplasm would activate the cGAS-STING pathway to induce innate immune responses.

Although cGAS specifically senses cytoplasmic DNA, activation of the cGAS-STING pathway has been observed with RNA viruses. Infection with human immunodeficiency virus (HIV), which has an RNA genome, has been shown to activate cGAS^5, 6^ although this is likely due to the production of reverse transcribed cDNA^5^. More recently, a study by Schoggins *et al*. suggests that +ssRNA viruses may be capable of activating the cGAS-STING pathway without reverse transcription of its genome^7^. The investigators observed that expression of cGAS via lentiviral transduction in STAT1^−/−^ fibroblasts was capable of further increasing expression of a wide array of ISGs that function as part of a greater antiviral response against +ssRNA viruses, including DENV and West Nile virus. It is thus curious how cGAS would be activated in such instances, as RNA is not an inducer of cGAS activity^6^. Determining how +ssRNA viruses trigger cGAS could provide insights into an area of the innate immune response that is presently not well understood.

In this study, we examined how DENV induces cGAS signaling by developing upon our recently reported finding that an attenuated DENV-2 strain, PDK53, robustly induces IFN-I^8^. Indeed, a robust and early IFN-I response contains the spread of PDK53 on a cell monolayer, thereby restricting its plaque size. Silencing of signaling intermediates in the IFN-I pathway, specifically IRF3, resulted in an increased number of PDK53 plaque forming units as well as plaque diameter^8^. Curiously, the plaque size of PDK53 remains small even when the assay is carried out in Vero or baby hamster kidney (BHK21) cells, which are dysfunctional in IFN-I signaling and production^8–10^. Non-secretory mechanisms of intercellular antiviral signaling, such as those induced by cGAS, could thus be important in regulating PDK53 plaque formation, which is often used to select for viral strains for development into live attenuated vaccine candidates. This hypothesis is further supported by the finding that PDK53 remains attenuated even in AG129 mice, which are deficient in IFNα/β/γ receptor signaling^11^. Here, we show that cGAS contributes to an IFN-I-independent antiviral response and suggest an explanation for how a +ssRNA virus is capable of triggering a DNA-specific sensor.

## Results

### cGAS-mediated antiviral signaling is activated during DENV infection and is spread from infected cells to neighboring uninfected cells via gap junctions

**Figure 1:**
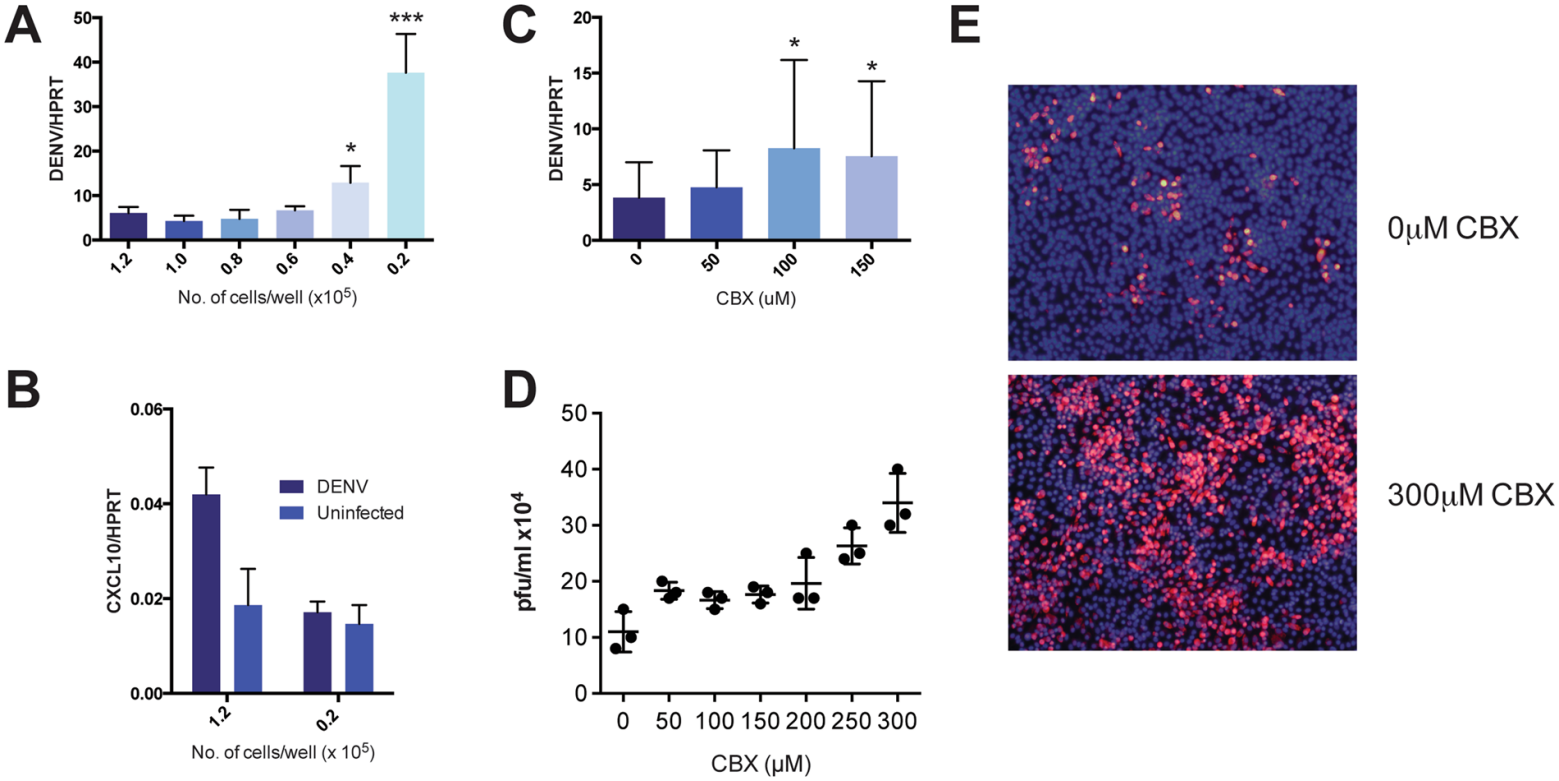
DENV-infected BHK21 activates ISGs in neighboring cells via gap junctions. (**A**) BHK21 cells, seeded with increasing proximal distance, were infected with DENV2-PDK53 at MOI 1 for 24 hrs before DENV infection was measured by DENV RNA detection. Results are normalized to *HPRT* expression and represent mean ± SD of at least two independent experiments. (**B**) BHK21 cells were seeded at either 1.2 × 10^5^ or 2 × 10^4^ cells per well and either uninfected or infected with DENV2-PDK53 at MOI 1 for 24 hrs. *CXCL10* expression was then measured via RT-qPCR and normalized to *HPRT* expression. (**C**,**D**) BHK21 cells were treated with increasing concentration of CBX before infection with DENV2-PDK53 at MOI 1 for 24 hrs. Viral load was assessed by detection of DENV viral RNA with RT-qPCR (**C**) and plaque assay (**D**). RT-qPCR results are normalized to *HPRT* expression and represent mean ± SD of at least seven independent experiments. Plaque assay results shown represent triplicate samples. (**E**) Spreading of DENV2-PDK53 at 72 hrs post infection in BHK21 cells treated with 300 μM CBX was assessed using immunofluorescent staining of DENV E-protein (red) and nuclei (DAPI, blue). All data are represented as mean ± SD, and *depicts P < 0.05 and ***depicts P < 0.001.

The plaque size of the DENV-2 PDK53 strain is limited by the heightened antiviral state in uninfected cells that surround foci of infected cells^8^. We thus first asked how physical contact between cells affect the outcome of DENV infection. We seeded BHK21 cells at decreasing cell count (Supplementary Fig. S1–4), infected with the same multiplicity of infection (MOI) of one and measured viral replication by quantitative RT-PCR (qRT-PCR). Results showed an inverse relationship between cell density and viral replication; reduced cell seeding resulted in increased PDK53 replication (Fig. 1A). In addition, the expression level of *CXCL10*, which has been shown to be upregulated during DENV infection^12^, was reduced with lower cell count (Fig. 1B). This suggests that increasing the distance between cells reduces their ability to transfer danger signals, possibly through gap junctions, thereby exacerbating DENV infection.

To test the role of gap junctions in the intercellular transfer of antiviral signals, we chemically blocked gap junctions of BHK21 cells with carbenoxolone (CBX)^13^ and then infected these cells with PDK53. A dose-dependent increase of DENV RNA (Fig. 1C) and plaque titers (Fig. 1D) with increasing concentrations of CBX at nontoxic levels were observed (see Supplementary Fig. S5). Immunofluorescent staining of DENV envelope (E) protein 72 hrs post-infection also showed that, compared to control cells without CBX (Fig. 1E, upper), CBX treatment increased the spread of DENV infection in the cell monolayer (Fig. 1E, lower) when incubated in a carboxymethylcellulose supplemented medium. These results collectively indicate that the optimal host defence against DENV requires the presence of functional gap junctions, through which cGAS induced cGAMP is known to spread from cell to cell^14^.

### DENV infection activates cGAS and subsequent cGAMP production in human A549 cells

**Figure 2:**
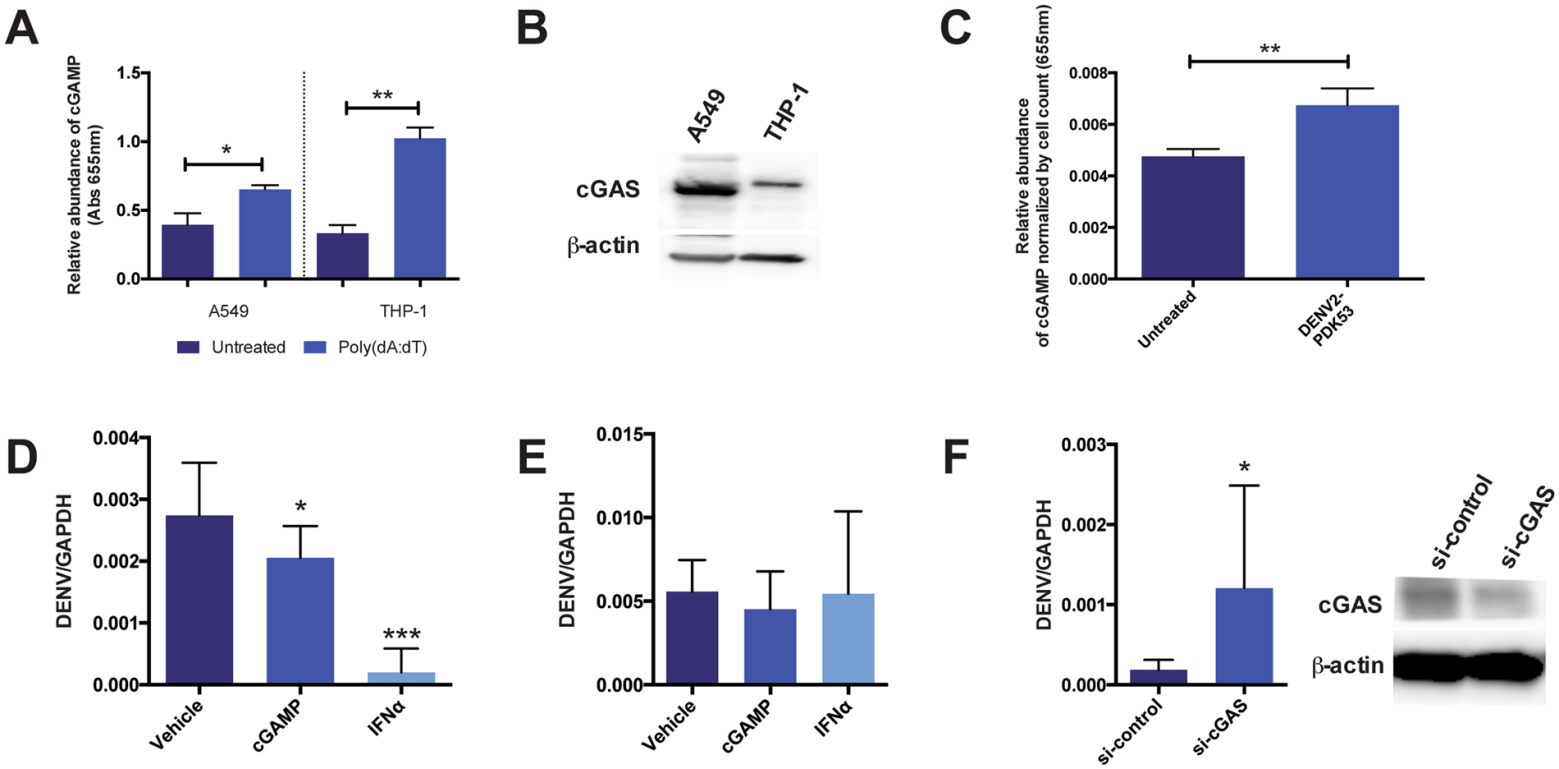
DENV infection activates cGAS-dependent antiviral responses. (**A**) *In vitro* assay for functional cGAS expression in A549 and THP-1 cell lysates as indicated by the production of cGAMP in the presence of 1 μg/ml poly(dA:dT). Results demonstrate that cGAS is functional in A549 as it is in THP-1, as shown previously^3^. (**B**) Western blot analysis of cGAS expression in A549 and THP-1 cells (control). THP-1 cells used as positive control and not as point of comparison for cGAS expression levels between A549 and THP-1 cell lines. (**C**) cGAMP production in A549 cells that are uninfected or infected with DENV2-PDK53 at MOI 1 for 24 hrs. Absorbance values normalized by cell count of each group. Results shown are representative of at least three independent experiments. (**D**,**E**) DENV RNA levels in infected A549 cells that were pre-treated (**D**) or post-treated (**E**) with 1 μg/ml cGAMP or 250 units/ml IFNα. Results are normalized by *GAPDH* expression and represent mean ± SD of at least four independent experiments. (**F**) DENV RNA levels in cGAS knockdown (si-cGAS) A549 cells compared to siRNA scrambled control (si-control) when infected with DENV2-PDK53 at MOI 1 for 24 hrs, assessed by RT-qPCR and normalized by *GAPDH* expression. Western blot confirmation of decreased cGAS expression in si-cGAS A549 cells, normalized with β-actin. Data is represented as mean ± SD of at least three independent experiments. In this figure, *depicts P < 0.05; **depicts P < 0.01 and ***depicts P < 0.001.

To determine if PDK53 infection activates cGAS in cells, the human lung carcinoma cell line, A549, was used as it forms effective gap junctions^15^ and is permissive to DENV infection^16^. The A549 cell line expresses functional cGAS that is capable of producing cGAMP in the presence of poly(dA:dT), ATP, and GTP when compared to THP-1 cells, a known cGAS-expressing human monocytic cell line^3^ (Fig. 2A,B). Likewise, PDK53 infection in A549 cells also resulted in increased amount of cGAMP production compared to uninfected cells (Fig. 2C).

### Activation of the cGAS-mediated antiviral program prior to DENV infection leads to decreased viral replication

Multiple studies have shown that IFN-I pre-treatment results in cells that are refractory to DENV infection, while the virus is less resistant to IFN treatment after infection is established^1, 17–19^. To determine the effects of cGAMP on DENV infection, we treated A549 cells with cGAMP before or after the establishment of DENV infection. Pre- and post-infection treatments with IFNα were included as controls, which as expected, strongly inhibited DENV infection when treatment was initiated pre- but not post-infection (Fig. 2D,E). Likewise, pre- but not post-infection treatment of cells with cGAMP significantly reduced viral RNA in infected cells although the degree of difference was smaller compared to IFNα pre-treatment (Fig. 2D,E).

To confirm a functional role of cGAS in the host defense against DENV, we measured the level of DENV infection after siRNA-mediated silencing of cGAS expression in A549 cells. At 24 hrs post-infection, increased levels of DENV RNA in cGAS-silenced A549 cells were detected compared to cells transfected with scrambled siRNA control (Fig. 2F). These results thus indicate that cGAS activation and the spread of cGAMP to neighboring uninfected cells could contribute to limiting the spread of PDK53 infection.

### DENV infection leads to release of mitochondrial DNA into the cytosol that may activate cGAS

**Figure 3:**
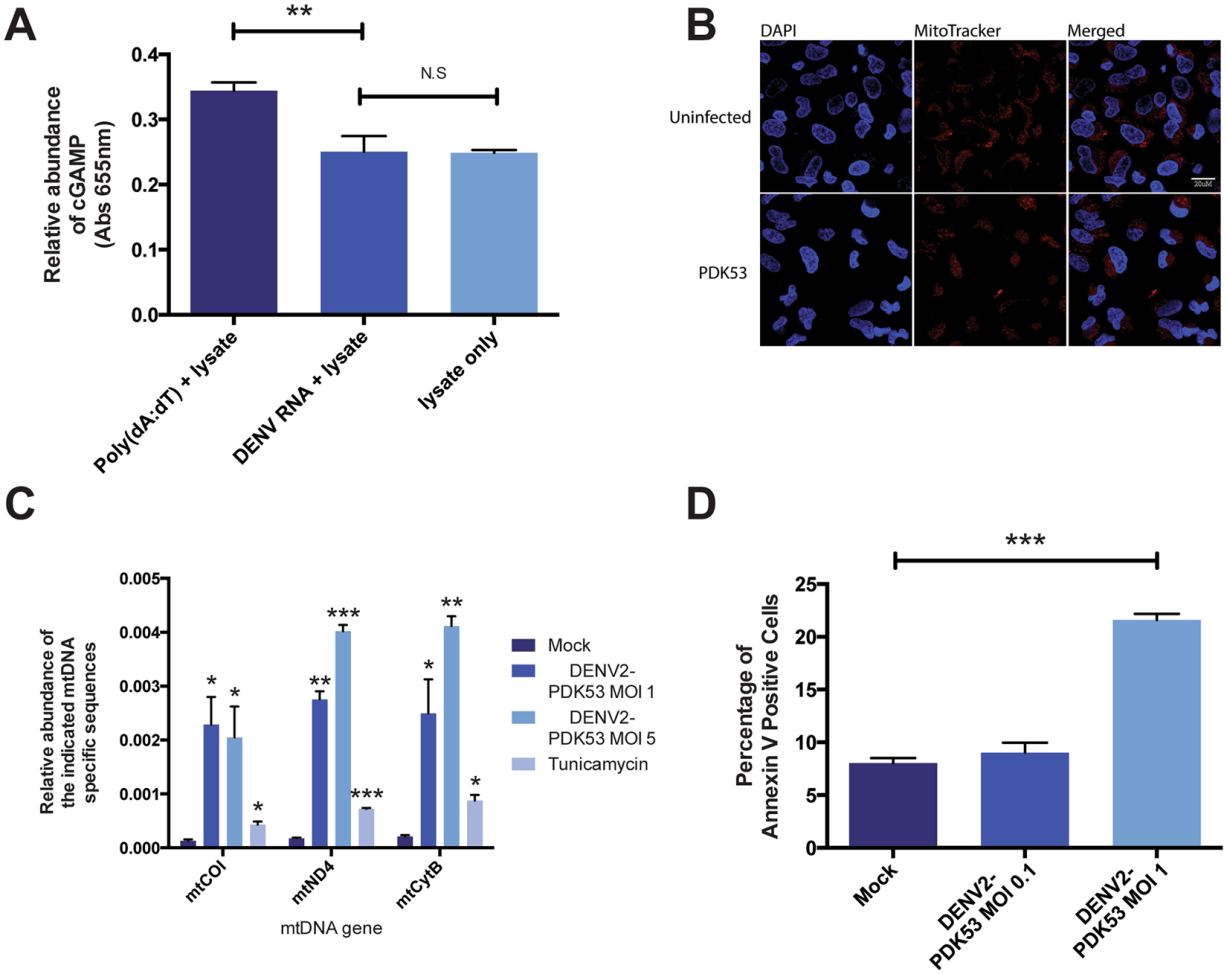
DENV infection activates cGAS through the release of mtDNA into the cytosol. (**A**) cGAMP activity measured in A549 lysates incubated with 1 μg/ml DENV-derived RNA or 1 μg/ml poly(dA:dT). (**B**) Cellular localization of mitochondria (red) in A549 cells infected with DENV2-PDK53 at MOI 1 for 72 hrs. (**C**) Fold induction of levels of mitochondria-specific DNA sequences *mtCOI*, *mtND4*, and *mtCytB* present in the cytosol of A549 cells infected with DENV2-PDK53 at MOI 1 and MOI 5 or treated with 4 μg/ml tunicamycin. (**D**) Percentage of Annexin V positive cells in A549 cells infected with DENV2-PDK53 at MOI 0.1 or MOI 1 for 48 hrs, assessed by flow cytometry. In this figure, *depicts P < 0.05; **depicts P < 0.01 and ***depicts P < 0.001.

Given that cGAS plays a role in the antiviral response to PDK53 infection, we next sought to determine how an RNA virus activates a cytoplasmic DNA sensor. First, we confirmed that in our experimental system, cGAS is not activated by RNA^6^. We stimulated cGAS in A549 cells with DENV-derived RNA or poly(dA;dT) and then measured subsequent cGAMP production using a previously described protocol^20^. In agreement with previous work^6^, our results showed no significant change in cGAMP levels with DENV RNA (Fig. 3A).

We next postulated that mitochondrial DNA (mtDNA) could be a ligand that activates cGAS as it was recently shown that mtDNA but not viral DNA primes antiviral innate immune responses against herpesvirus^21^. We thus tested for the possibility that mitochondrial damage occurs during PDK53 infection. Using MitoTracker Deep Red dye staining coupled with confocal immunofluorescence microscopy, we observed mitochondrial aggregate formation in PDK53-infected A549 cells at 24 hours post infection compared to uninfected control cells (Fig. 3B). Using a cell fractionation approach, we measured mtDNA in the cytoplasmic fraction of A549 cells infected with DENV. We observed a MOI-dependent increase in mtDNA in the cytoplasm of infected compared to uninfected cells (Fig. 3C). Additionally, as mitochondrial damage often precedes the induction of apoptosis^22^, we compared levels of apoptosis in PDK53-infected against uninfected cells using Annexin V staining. As expected, we observed a significant increase in Annexin V-stained cells 48 hrs post-infection (Fig. 3D). Collectively, these results indicate that mitochondrial alteration or damage upon DENV infection releases mtDNA into the cytoplasm of infected cells that may contribute to the activation of cGAS signaling in response to +ssRNA virus infection.

## Discussion

Our investigations reveal a non-canonical mechanism through which DENV activates a cytosolic DNA specific sensor, cGAS. Specifically, we show that DENV infection could result in mtDNA release into the cytosol that, in turn, likely triggers a cGAS-mediated antiviral response.

The longstanding paradigm regarding cellular antiviral mechanisms in the setting of DENV infection focuses almost exclusively on RNA sensors such as RIG-I and MDA5^23–26^. In this model, RNA viruses directly bind to RIG-I and activate downstream IRF3 signaling, thereby increasing IFN-I production^27^. However, as demonstrated in a recent study^7^, the DNA sensor cGAS likely mediates an antiviral response found to strongly inhibit +ssRNA virus replication. Furthermore, *cGAS* ^*−/−*^ mice showed increased rates of infection and mortality compared to *wt* mice when infected with +ssRNA West Nile virus^7^. These findings, along with ours, raise a caveat in interpreting PRR activation in innate immunity studies.

The activation of cGAS, however, is still due to its specificity as a DNA sensor. Indeed, a recent study by Aguirre *et al*.^28^ also demonstrated that mtDNA is released into the cytosol to activate cGAS during DENV-2 infection. Our findings thus corroborate their findings and collectively indicate a role for cGAS in inducing IFN-I during DENV infection.

Interestingly, Aguirre *et al*. used the DENV-2 16681 strain in their study^28^. PDK53 was derived from 16681 through 53 serial passages in primary dog kidney cells^29^. We have recently shown that PDK53 induces a stronger innate immune response than its parental 16681 strain^8^. However, none of the mutations that resulted in the attenuated phenotype of PDK53 were found in the NS2B gene^30^. This lack of genetic difference in NS2B despite the ability of PDK53 to induce IFN-I more robustly than 16681 is thus intriguing, especially given the NS2B-mediated protease activity in destroying cGAS^28^. These findings suggest that PDK53 infection, which replicates its RNA genome in infected cells more rapidly than 16681 does^8^, may overactivate the innate immune and metabolic responses during infection. The resulting cellular damage then produces greater levels of mtDNA-dependent cGAS activation than can be effectively inhibited by the expressed levels of NS2B. Further studies will be needed to test this possible explanation.

Our *in vitro* findings also complement previously reported clinical observations. A prospective study of dengue fever patients has revealed changes in the serum metabolome and lipidome suggestive of mitochondrial dysfunction, such as disruptions in the beta-oxidation pathway of fatty acids^31^. Likewise, measurement of cell-free DNA isolated from plasma showed higher quantity of mtDNA in dengue patients compared to uninfected healthy control subjects^32^. These studies, combined with our mtDNA data, therefore suggest a role for the mitochondria in response to DENV infection and perhaps also live attenuated virus vaccination, given that our experiments were conducted using PDK53. Nevertheless, how mtDNA release is mediated mechanistically during infection is still not fully understood^33, 34^. A potential mechanism could involve Bax and Bak, two apoptotic proteins that form pores to normally facilitate cytochrome c release but may also allow for passage of mtDNA into the cytoplasm^33–36^. Additionally, DENV NS4B protein could also trigger mitochondrial elongation, a process that may enable mtDNA release into the cytosol^37^. Chatel-Chaix *et al*. has also shown how DENV infection leads to decreased phosphorylation of dynamin-related protein 1 (Drp1) at serine residue 616, thereby lowering Drp1 activity^37^. As Drp1 silencing has been shown to result in mitochondrial elongation and mtDNA release into the cytosol^38^, it is plausible that reduced Drp1 activity upon DENV infection enables mtDNA release that activates cGAS and cGAMP production.

In conclusion, our findings indicate a functional role for the DNA sensor, cGAS, in triggering antiviral responses that limit the spread of the DENV-2 PDK53 vaccine strain from infected to uninfected cells.

## Materials and Methods

### Cell lines and Reagents

BHK21 (ATCC CCL10) and A549 (ATCC CCL185) cells were purchased from the American Type Culture Collection (ATCC) and were cultured in RPMI 1640 (Gibco 11857093) and DMEM media (Gibco 11995065) supplemented with 9% fetal calf serum (HyClone SH30071), respectively. THP1-Blue^TM^ ISG cells used for cGAMP *in vitro* assay was purchased from Invivogen and cultured per manufacturer’s recommendation. Reagent stocks including carbenoxolone (Sigma-Aldrich C4790), poly(dA:dT) (Invivogen tlrl-patn), cGAMP (Invivogen tlrl-nacga23), human recombinant IFNα (Abcam ab48750), tunicamycin (Sigma-Aldrich T7765), and MitoTracker Deep Red (ThermoFisher M22426) were stored at −20 °C until use. Endotoxin free h3H5 chimeric human/mouse antibodies against DENV E-protein (h3H5) were constructed as previously described^39^. Anti-human cGAS antibody (HPA031700) was purchased from Sigma-Aldrich. APC Annexin V stain was purchased from BD Pharmingen (550474).

### Virus Stock

DENV2-PDK53 (GenBank Accession Number KU725664) is a vaccine strain developed by the Mahidol University Center for Vaccine Development (a gift from Claire Huang, Centers for Disease Control and Prevention). DENV2-PDK53 was propagated in C6–36 cells, harvested 96 hrs post-infection, and purified through 30% sucrose. Virus pellets were resuspended in 5 mM HEPES, 150 mM NaCl, and 0.1 mM EDTA (HNE) buffer, and were then stored at −80 °C until use. Infectious titers for the virus were determined through plaque assays as previously described^40^.

### Two-step RT-Quantitative Polymerase Chain Reaction (RT-qPCR)

RNA was extracted from cells using the RNeasy Mini kit (Qiagen 74106). Total RNA was reverse transcribed into cDNA using iScript Reverse Transcriptase (Bio-Rad 1708891). cDNA samples were then amplified by use of LightCycler 480 SYBR Green I (Roche 04707516001) with primers for pan-serotype DENV, BHK21 hypoxanthine-guanine phosphoribosyltransferase (*HPRT*), BHK21 C-X-C motif chemokine ligand 10 (*CXCL10*), human mitochondrial cytochrome c oxidase subunit I (*mtCOI*), human mitochondrial NADH dehydrogenase subunit 4 (*mtND4*), human mitochondrial cytochrome B (*mtCytB*), or human glyceraldehyde 3-phosphate dehydrogenase (*GAPDH*) (see Supplementary Table S1 for primer sequences). All primers were obtained from AITbiotech. RT-qPCR was performed using a LightCycler 480 RT-qPCR system (Roche) and analyzed with LightCycler 480 Software (Roche Diagnostics).

### Gap junction inhibition

To inhibit gap junction functions, BHK21 cells were incubated with varying concentrations between 50–300 μM of CBX (Sigma-Aldrich C4790) for 3hrs in media at 37 °C. The treated cells were washed extensively with PBS before virus infection to decrease the chance of CBX in the media interfering with the infection process.

### MTS Assay

CellTiter 96 AQueous One Solution Cell Proliferation Assay (MTS) was purchased from Promega (G3580). To assay effects of CBX, BHK21 or A549 cells were seeded overnight prior to treatment with CBX for 3 hrs. Cells were subsequently washed with PBS (to decrease the chance of CBX in the media interfering with infection, and incubated for 24 hrs before MTS was added and absorbance read at 490 nm.

### Immunofluorescence and Confocal Microscopy

CBX-treated BHK21 cells were infected with DENV2-PDK53 at MOI 0.01. Virus inoculum was subsequently replaced with 0.8% methylcellulose in 3% fetal calf serum supplemented media. After 72 hrs, the cells were fixed with 3% paraformaldehyde for 30 mins at room temperature and permeabilized with 0.1% saponin for 30 mins at room temperature in a moist chamber. DENV detection was visualized by staining with human anti-DENV E protein antibody (h3H5) followed by Alexa Fluor 555 goat anti-human antibody (Life Technologies A-21433). Processed cells were viewed using an Olympus U-LH100HG microscope at 4x magnification. To visualize the formation of mitochondria aggregates in DENV infected A549 cells, infected cells were stained with MitoTracker Deep Red FM (ThermoFisher M22426) according to manufacturer’s instructions and visualized using the LSM 710 confocal microscope (Carl Zeiss) at 63x magnification.

### *In vitro* cGAMP Activity Assay

cGAMP activity was assessed as previously described^20^. Briefly, for relative quantification of cGAMP that was produced in cells during DENV infection, cell lysates were prepared by freeze-thawing five times. The lysate was subsequently treated with 1 unit/μl of benzonase (Sigma-Aldrich E1014) at 37 °C for 30 mins before heat treatment at 95 °C for 5 mins. The supernatant was collected after centrifuging lysates at 22000 g for 10 mins. The relative amounts of cGAMP between samples were measured by applying the lysates to THP-Blue ISG (Invivogen) cells, and ISG activation was detected with Quantiblue reagent (Invivogen rep-qb1). The lysates were introduced into THP1-Blue ISG (Invivogen) cells through digitonin (Sigma-Aldrich D141) permeabilization. Cells were subsequently incubated with 10x digitonin permeabilization buffer (50 mM HEPES pH7.0, 100 mM KCl, 3 mM MgCl_2_, 85 mM sucrose, 0.2% BSA, 10 μg/ml digitonin, 0.1 mM DTT and 1 mM ATP) together with A549 cell lysate for 30 mins at 37 °C and subsequently replaced with media.

### Determination of cGAS Function and Specificity of DNA Recognition

To determine the expression of functional cGAS, cell lysates were incubated for 45 mins at 37 °C with poly(dA:dT), GTP, and ATP diluted in buffer composed of 20 mM HEPES pH7.2, 5 mM MgCl_2_, and 0.1 mM EGTA (or buffer only as negative control). The resulting cell lysate was subsequently used to assess for cGAMP content as described above. To assay the DNA-specific nature of cGAS, the same protocol above was followed, with either poly(dA:dT), DENV-derived RNA, or buffer only conditions.

### siRNA-Mediated Gene Silencing

Human cGAS and scrambled siRNA were obtained from SABio. Human cGAS (5′-GCCUUCUUUCACGUAUGUA, Antisense: UACAUACGUGAAAGAAGGC) and scrambled siRNA duplexes (5 nM, SN001-10D) were incubated with Lipofectamine RNAiMAX Transfection Reagent (Invitrogen 13778150) in serum-free media for 20 mins at room temperature and added dropwise to A549 cells. Transfection of siRNA was performed twice (0 hrs and 72 hrs), and infection with DENV was performed 24 hrs after the second transfection.

### Western Blotting

Protein lysates of A549 or THP-1 cells were isolated in NP-40 buffer with protease inhibitor at 1:100 concentration (Sigma-Aldrich P8340). Lysates were then run in 4–15% Mini Protean TGX gradient gels (Bio-Rad). After transferring proteins to a PVDF membrane (Millipore), the membrane was blocked in 5% BSA + 0.5% Tween in PBS for 1 hr at room temperature. The membrane was then probed with primary antibody overnight at 4 °C. Primary antibodies included human cGAS (Sigma-Aldrich HPA031700, 1:500) and human β-actin (Cell Signaling 8H10D10, 1:1000). The membrane was then probed with secondary antibody for 45 mins at room temperature. Secondary antibodies included HRP-conjugated anti-rabbit (Abcam 6721) (1:10000) and HRP-conjugated anti-mouse (Dako P0447, 1:20000). To minimize background signal, the membrane was washed with PBS and 0.5% Tween (PBS-T) for 5 mins, four times each, before being treated with Amersham ECL prime western blotting detection reagent (GE RPN2232) and imaged with ImageQuant LAS 4000 (GE).

### Cytosolic Mitochondria DNA Detection

To detect the presence of mitochondrial DNA in the cytosol, the cytoplasm was isolated by a series of centrifugation steps on A549 cell lysates^21, 41^. A549 cell lysates were first isolated by lysing cells with a buffer containing 150 mM NaCl, 50 mM HEPES pH 7.4, and 20 μg/ml digitonin. Cells were incubated in the buffer for 10 mins at 4 °C using end-to-end rocking. The cells were then passed through a 31-gauge needle ten times to complete the cell lysis. The supernatant was then centrifuged at 1000xG for 10 mins and then 20000xG for 20 mins to isolate the cytosol. DNA was subsequently extracted from the resulting supernatant with the QIAamp DNA Mini Kit (Qiagen) according to manufacturer’s instructions. RT-qPCR analysis was performed to measure mtCOI, mtND4 and mtCytB in the unfractionated whole cell extract and the cytosolic fraction samples. The relative expression of the various genes was determined by a previously described method^21^. Briefly, the Cp values were obtained by having the unfractionated samples serve as the normalization control for the cytosolic fraction.

### Annexin V Staining

A549 cells were infected with DENV2-PDK53 at MOI 1 and MOI 5 for 48 hrs.

APC Annexin V staining (eBioscience A35110) was performed using manufacturer protocol, with positively stained Annexin V cells detected via FACS. Flow cytometry was performed on the BD LSRFortessa flow cytometer and analyzed with FACSDiva software.

### Preparation of *in vitro* Transcribed DENV RNA

*In vitro* transcribed full length DENV2 RNA was prepared using the following method. Plasmid containing an infectious clone of the DENV2 strain EDEN 3295 (Genbank accession EU081177) was linearized at the end of the dengue 3′UTR. The linearized plasmid was purified using the standard phenol:chloroform:isoamyl alcohol (Sigma-Aldrich) method, followed by precipitation with sodium acetate and ethanol. RNA was produced using the Ambion MegaScript T7 kit (Life Technologies AM1334), following manufacturer’s instructions, with the exception of adding an additional 40U RNasin Ribonuclease Inhibitor (Promega N2111) to the *in vitro* transcription mix. After the end of *in vitro* transcription, the mix was treated with 4U of TURBO DNase (Life Technologies AM2238) per 1 μg input of plasmid to ensure complete digestion of DNA. The *in vitro* produced RNA was recovered using the lithium chloride precipitation method^42^ and the RNA pellet was resuspended in nuclease-free water. Concentration was quantified with the NanoDrop 2000 Spectrophotometer (ThermoFisher), and RNA was stored at −80 **°**C until use.

### Statistics

Error bars in figures represent mean ± SD, and the Student’s t-test was used to compare significant differences. *depicts P < 0.05; **depicts P < 0.01 and ***depicts P < 0.001. Data shown are the representative of at least two or three independent experiments unless otherwise stated. Statistics were performed using GraphPad Prism v6.0 software.

## Electronic supplementary material

Supplementary Figures
