# Supplementary material for: Dengue virus activates cGAS through the release of mitochondrial DNA
Source: Sci Rep. 2017 Jun 15;7:3594. doi: 10.1038/s41598-017-03932-1 (PMC5472572; doi:10.1038/s41598-017-03932-1)
Supplement: Supplementary file 1 — Supplementary Figures [file 41598_2017_3932_MOESM1_ESM.pdf]

Dengue virus activates cGAS through the release of mitochondrial DNA

Bo Sun[1,2], Karin B. Sundström[1], Jun Jie Chew[1], Pradeep Bist[1], Esther S. Gan[1], Hwee Cheng Tan[1], Kenneth C. Goh[1], Tanu Chawla[1], *Choon Kit Tang[1], *Eng Eong Ooi[1,3,4,5]

[1]Programme in Emerging Infectious Diseases, Duke-NUS Medical School, Singapore 169857

[2]Duke University School of Medicine, Durham, NC, 27710 USA

[3]Department of Microbiology and Immunology, Yong Loo Lin School of Medicine, National University of Singapore, Singapore

[4]Saw Swee Hock School of Public Health, National University of Singapore, Singapore

[5]Singapore MIT Alliance in Research and Technology, Infectious Diseases Interdisciplinary Group, Singapore

*Correspondence to:

Choon Kit Tang (cktang79@gmail.com) or

Eng Eong Ooi (engeong.ooi@duke-nus.edu.sg)

Supplementary Table S1. Sequences of RT-qPCR primers utilized.

Gene Target	Primer Sequence
DENV LYL 3'UTR	Forward: TTGAGTAAACYRTGCTGCCTGTAGCTC<br>Reverse: GAGACAGCAGGATCTCTGGTCTYTC
HPRT (BHK21)	Forward: GATAGATCCACTCCCATAACTG<br>Reverse: TACCTTCAACAATCAAGACATTC
CXCL10 (BHK21)	Forward: TCTGAATCCAGAGTCTGAGG<br>Reverse: AGGGACAATTAGGAGTAGCC
mtCOI (human)	Forward: ATTCATCGGCGTAAATCTAA<br>Reverse: AGGCTTCTCAAATCATGAAA
mtND4 (human)	Forward: CCTCGTAGTAACAGCCATTC<br>Reverse: TTGAAGTCCTTGAGAGAGGA
mtCytB (human)	Forward: CCATCCTCCATATATCCAAA<br>Reverse: CCAATGATGGTAAAAGGGTA
GAPDH (human)	Forward: GAGTCAACGGATTTGGTCGT<br>Reverse: TTGATTTTGGAGGGATCTCG

S1

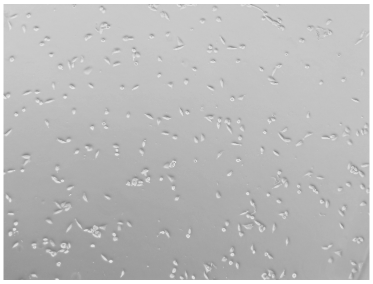

2 x 10^4 cells/well

S2

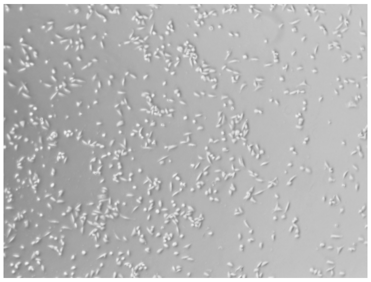

4 x 10^4 cells/well

S3

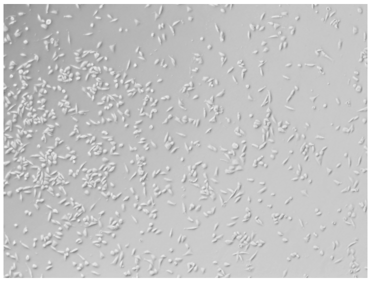

6 x 10^4 cells/well

S4

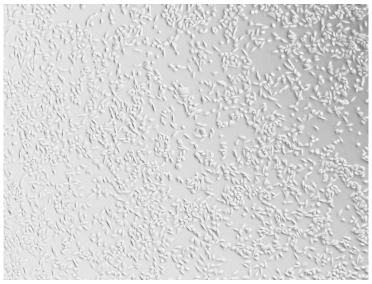

1.2 x 10^5 cells/well

S5

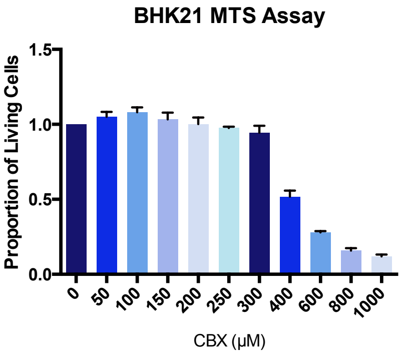

Supplementary Figures. (S1-4) Representative images of 2 x 10^4, 4 x10^4, 6 x 10^4, 1.2 x10^5 BHK21 cells plated in a single well of a 24-well plate after overnight incubation. **(S5)** MTS assay performed for CBX treatment of BHK21 cells; CBX treatment performed for 3hrs at concentrations shown before removal of drug and further incubation of cells for 24hrs.
